# Phew! time to focus on physical health and wellbeing: improving the assessment and management of physical health in an early intervention in psychosis service

**DOI:** 10.1192/bjo.2021.487

**Published:** 2021-06-18

**Authors:** Bethany Cole, Emma Bailey, Liz Ewins

**Affiliations:** Avon and Wiltshire Mental Health Partnership NHS Trust

## Abstract

**Aims:**

NICE guidelines recommend that patients under Early Intervention (EI) in Psychosis Services have systematic monitoring and intervention of cardiometabolic risk factors. We undertook a Quality Improvement Project (QIP) in the Bath and North East Somerset (BaNES) EI Team to improve rates of compliance with national guidelines. We aimed to increase the percentage of service users with a physical health assessment documented in the past 12 months. Other aims included improving monitoring of physical health parameters in those taking antipsychotic medication and increasing the delivery of interventions for abnormal results.

**Background:**

The most common cause of premature mortality in people who experience psychosis and schizophrenia is cardiovascular disease. The 'Standards for Early Intervention in Psychosis Service' states that patients should be offered personalised healthy lifestyle interventions, including advice on diet, physical activity, and access to smoking cessation services. Physical health should be monitored at least annually, with more frequent assessments if antipsychotic medication is prescribed.

**Method:**

We identified seven key factors for improving physical health: Body Mass Index (BMI), Blood Pressure, Glucose Regulation, Blood Lipids, Smoking, Alcohol and Illicit drug use. Baseline compliance and intervention rates were measured in March 2019. Six ‘Plan, Do, Study, Act’ Cycles were completed over the following ten months. Examples of the changes made included: a new online diary and whiteboard, abbreviation of the assessment form, teaching for the EI team, and a new weekly ‘Physical Health and Wellbeing’ (PHeW) Clinic. This clinic involved phlebotomy, discussions around lifestyle choices, review of medication side effects, and neurological examination.

We measured the compliance with guidelines each month and the total number of interventions delivered at three-monthly intervals. We collected qualitative feedback on these changes in team meetings and with written questionnaires (including feedback from patients).

**Result:**

Documentation of all key factors doubled from 30.2% at baseline to 63.3% in January 2020. The total number of interventions for raised BMI and lipid levels also increased. Feedback from staff and patients was positive. The clinic helped start conversations with patients about lifestyle choices, prompting improvements in weight, physical activity, lipid levels, and alcohol intake. Patient awareness and ownership over their physical health also improved.

**Conclusion:**

This project utilised multiple strategies to reduce health complications for BaNES EI service users. A structural change in the assessment and management of physical health proved to be an effective and sustainable solution to optimise the health and wellbeing of this patient group.

